# Cytokine Networks Dysregulation during HTLV-1 Infection and Associated Diseases

**DOI:** 10.3390/v10120691

**Published:** 2018-12-05

**Authors:** Nicolas Futsch, Gabriela Prates, Renaud Mahieux, Jorge Casseb, Hélène Dutartre

**Affiliations:** 1 Équipe Oncogenèse Rétrovirale, Equipe Labellisée «FRM», CIRI—Centre International de Recherche en Infectiologie, Université Claude Bernard Lyon 1, Inserm U1111, CNRS UMR5308, Labex Ecofect, ENS Lyon, F-69007 Lyon, France; nicolas.futsch@ens-lyon.fr (N.F.); renaud.mahieux@ens-lyon.fr (R.M.); 2 Institute of Tropical Medicine of São Paulo, São Paulo, SP 05403-000, Brazil; gabrielaprates12@hotmail.com (G.P.); jcasseb10@gmail.com (J.C.); 3 Laboratory of Dermatology and Immunodeficiencies, Department of Dermatology, University of São Paulo Medical School, São Paulo, SP 01246-100, Brazil

**Keywords:** HTLV-1, immune system, HAM/TSP, ATLL, cytokines

## Abstract

Human T-cell leukemia virus type 1 (HTLV-1) is the causative agent of a neural chronic inflammation, called HTLV-1-associated myelopathy/tropical spastic paraparesis (HAM/TSP) and of a malignant lymphoproliferation, called the adult T-cell leukemia/lymphoma (ATLL). The mechanisms through which the HTLV-1 induces these diseases are still unclear, but they might rely on immune alterations. HAM/TSP is associated with an impaired production of pro-inflammatory cytokines and chemokines, such as IFN-γ, TNF-α, CXCL9, or CXCL10. ATLL is associated with high levels of IL-10 and TGF-β. These immunosuppressive cytokines could promote a protumoral micro-environment. Moreover, HTLV-1 infection impairs the IFN-I production and signaling, and favors the IL-2, IL-4, and IL-6 expression. This contributes both to immune escape and to infected cells proliferation. Here, we review the landscape of cytokine dysregulations induced by HTLV-1 infection and the role of these cytokines in the HTLV-1-associated diseases progression.

## 1. Introduction

Human T-cell leukemia virus type 1 (HTLV-1), the first oncogenic retrovirus identified in humans, is the etiological agent of two main diseases—HTLV-1-associated myelopathy/tropical spastic paraparesis (HAM/TSP) and the adult T-cell leukemia/lymphoma (ATLL). Both diseases generally occur after a long period of latency, with approximatively, 1 to 2% and 2 to 4% of carriers being at risk for HAM/TSP or ATLL development, respectively [1]. An increased HTLV-1 proviral load is observed both in ATLL and HAM/TSP patients [2]. In addition, both diseases seem not only to be driven by HTLV-1 itself but also through potential interactions between the virus and its host, although mechanisms leading to HAM/TSP or ATLL development, are not fully understood. HTLV-1 provirus is found in several cell types *in vivo*. This includes CD4+T and CD8+ T cells, neutrophils, monocytes, myeloid and plasmacytoids dendritic cells [3,4]. In vitro HTLV-1 infection has been reported in CD4+ and CD8+ T cells [5], myeloid and plasmacytoid dendritic cells [6], and monocytes-derived dendritic cells [3,7]. Although mainly analyzed in infected T-cells, HTLV-1 infection may perturb the ability of all these different cell types to secrete cytokines.

HTLV-1 is endemic in Japan, sub-Saharan Africa, the Caribbean region and South America (especially in Brazil), in addition to a small foci in the Middle East, Romania, and Australo-Melanesia [8]. Inter-individual transmission of HTLV-1 is the result of a direct exposure to infected cells which are present either in maternal milk, semen, or blood [1]. There seems to be a higher risk of developing HAM/TSP in the Caribbean and South America, while ATLL is the main HTLV-1-associated disease in Japan [8]. It has been suggested that the risk of ATLL development was mainly associated with a mother-to-child transmission of the virus [9], while the risk of HAM/TSP development results from infections through contaminated blood or sexual contacts [10,11]. However, the transmission route may not be the exclusive driver of disease onset, since ATLL cases were reported among patients with HAM/TSP [12]. In addition, HAM/TSP or ATLL diseases also occurred in young patients who had likely acquired the virus through breastfeeding [13].

HAM/TSP was defined as a progressive, chronic demyelination of the spinal cord, associated with an accumulation of both HTLV-1-infected cells and CD8+ T-cells, directed against HTLV-1 antigens, within the cerebrospinal fluid (CSF) and neural tissues [14,15,16,17]. ATLL is described as a highly aggressive HTLV-1-infected CD4+ T-cells proliferation and was classified into four clinical subtypes, i.e., smoldering, chronic, acute, and lymphoma subtypes [18].

Both diseases are characterized by an alteration in the immune system. While HAM/TSP is associated with a chronic inflammation, instrumental for the symptom’s manifestation, ATLL is characterized by an immunosuppressive state that favors the proliferation of the transformed HTLV-1-infected cells, thus, contributing to the malignancy. These different immune profiles are sustained by different cytokine networks, which may contribute to the specific HTLV-1-induced diseases development. However, the reasons for the disease occurrence in each HTLV-1 infected individual are still an important unanswered question. Markers of the disease evolution towards ATLL or HAM/TSP have not been identified. In this review, we try to explain how the interaction of the HTLV-1 with the immune system leads to a potential dysregulation of cytokines production or signaling, thus, allowing viral persistence and, eventually, pathogenesis.

## 2. Cytokines Supporting HTLV-1-Infected T-Cells Proliferation and Survival

**Figure 1:**
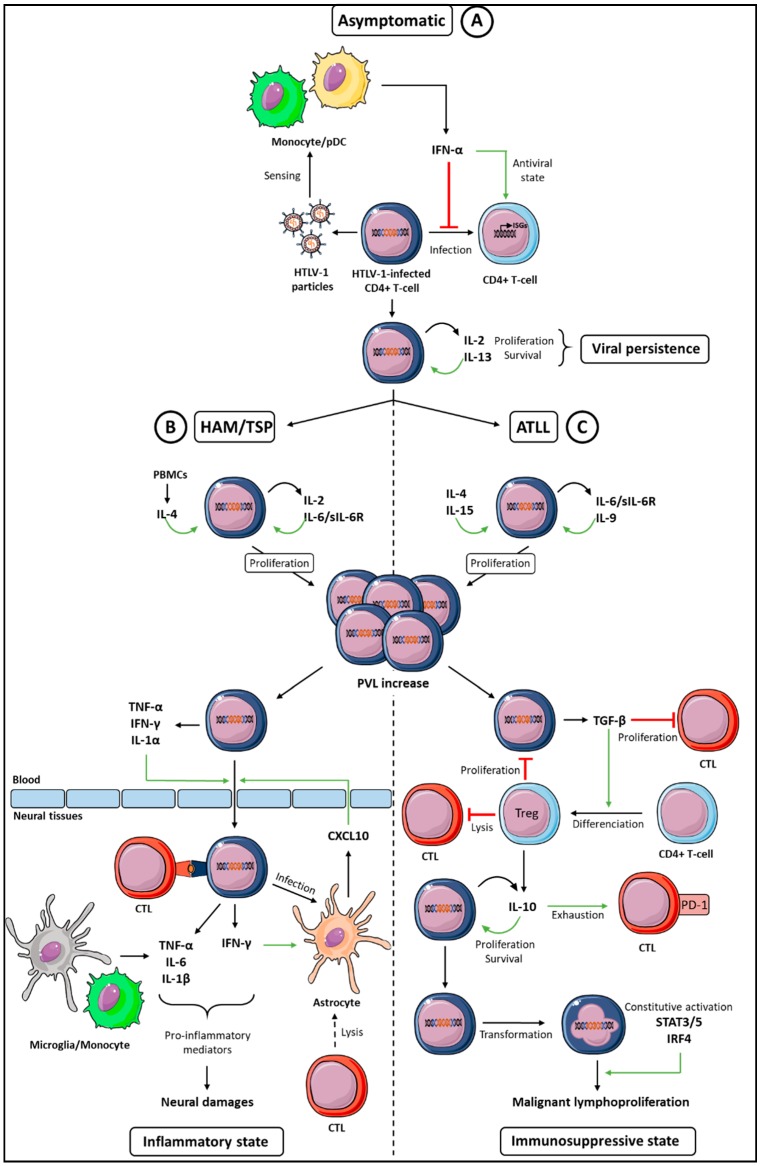
Global cytokine networks dysregulation during an HTLV-1 infection and HTLV-1-associated diseases. Three classes of cytokines can be involved in HTLV-1 persistence and diseases. (**A**) During the asymptomatic period that follows primo infection (**top**), IFN-α, IL-2, and IL-13 ensure, respectively, the antiviral control and proliferation of infected cells, both effects allowing viral persistence. (**B**) During the HAM/TSP disease (**left**), proinflammatory cytokines (IFN-γ, IL-1, TNF-α, and CXCL10) allow infiltration of the HTLV-1-infected T-cells in neural tissues that, in addition with IL-6, contribute to the chronic inflammatory state and neural damages. (**C**) During ATLL (**right**), immunosuppressive cytokines (TGF-β and IL-10) contribute to Cytotoxic T Lymphocytes (CTL) exhaustion, allowing an immunosuppressive state favorable to the malignant proliferation of HTLV-1-transformed cells. Green arrows indicate an increased function, red lines indicate a decreased function, and the curved arrow indicate autocrine proliferation. See text for details.

A hallmark of the HTLV-1-infected T-cells is their ability to proliferate independent of the TCR (T-cell Receptor) signaling, due to immortalization of the infected-cells This mainly requires activity of the Tax and the HBZ (HTLV-1 Basic Leucine Zipper Factor) viral proteins [19,20]. Survival and proliferation of the HTLV-1-infected cells are also supported by their dependence on several cytokines, such as IL-2, IL-4, IL-6, and IL-13 (Figure 1).

### 2.1. IL-2

IL-2 signaling induces cell proliferation and inhibition of apoptosis in human T-cells [21]. HTLV-1-infected cells isolated from asymptomatic carriers, HAM/TSP patients, and ATLL patients are all able to express the IL-2 receptor (IL-2R, CD25), mainly, because of the well-documented transcriptional effect of Tax on CD25 promotor [22]. However, only fresh HTLV-1-infected T-cells isolated from asymptomatic carriers and HAM/TSP patients express IL-2 mRNA [23], supporting an autocrine IL-2 proliferation loop in these individuals [24]. In line with this, it was reported that *in vitro* infected Peripheral Blood Mononuclear Cells (PBMCs) were dependent on IL-2, for their proliferation, until they get immortalized after several weeks in culture [25]. In these HTLV-1 infected T-cell lines, some characteristic of partial IL-2 independence, with constitutive JAK3/STAT3 phosphorylation, in the absence of IL2, was linked to the immortalization process. Consistently, leukemic cells from the ATLL patients, that are fully immortalized and transformed, are poorly or fully non-responsive to IL-2, for their proliferation [26,27,28], which could be associated with the low levels of IL-2 secreted by the HTLV-1-infected cell lines [29]. These studies suggest that the proliferation of leukemic cells *in vivo* could be partly IL-2 independent. Indeed, it has been reported that some HTLV-1-infected T-cells can proliferate without any addition of the exogenous IL-2 [29]. This IL-2-independent proliferation could result from a constitutive activation of the JAK/STAT (Janus kinases/Signal Transducer and Activator of Transcription) signaling [30], as exemplified by the constitutive phosphorylation of the STAT5 observed in IL-2-independent HTLV-1-infected T-cell lines [31]. However, this was observed in leukemic cells in only a small proportion of ATLL patients [31,32], suggesting that IL-2 dependent mechanisms could, nevertheless, contribute to the proliferation of the HTLV-1-infected cells in ATLL patients. Furthermore, CD25 expression on ATLL cells, may sequester IL-2, rather than induce IL-2 signaling, as could the soluble form of CD25, although, it was observed in humanized mice, infected by HTLV-1 [33]. In addition, IL-9 or IL-15, combined with IL-2, could better sustain the proliferation of PBMCs from chronic or smoldering ATLL patients, than IL-2 alone [34]. Interestingly, IL-9 expression is induced by both Tax and IL-2 [35], and the IL-15 receptor is expressed at the surface of leukemic cells, from the HTLV-1-infected patients [36]. Finally, the spontaneous proliferation of leukemic cells from chronic or smoldering ATLL patients is inhibited if they are sorted from the total PBMCs population [34]. Even though the proliferation of these isolated leukemic cells is not enhanced by IL-2 or IL-9 addition, it is restored after an interaction with autologous monocytes [34], thus, suggesting that *in vivo* leukemic cell proliferation may not only rely on cytokine loops but also on physical contacts with surrounding cells. Finally, a recent report showed that ATLL cell proliferation relies on the HBZ-induced BATF3 expression and BATF3/IRF4 network [37]. This further supports the fact that ATLL cells growth is not regulated through the IL-2 autocrine loop.

### 2.2. IL-4

IL-4 induces leukemic cells proliferation, when cells isolated from ATLL patients were grown *in vitro* [28,38]. This might be linked to a high expression of the IL-4 receptor (IL-4R), especially, at the surface of cells from acute ATLL patients [39]. IL-4 is undetectable in culture supernatants obtained from ATLL cells or in the supernatant from ATLL cells, before or after stimulation [38,40]. These results suggest that the HTLV-1 infection is not enough to maintain the IL-4 production and IL-4-induced proliferation. However, one cannot exclude that *in vivo* proliferation of the infected T-cells occurs within lymphoid organs, in which even low levels of IL-4 could act in an autocrine or paracrine manner.

IL-4 production may not be necessary to sustain the infected cell proliferation, if a constitutive IL-4 signaling is activated. Indeed, IRF-4 (Interferon Regulatory Factor 4) upregulation [41], could compensate the lack of IL-4 production by the HTLV-1-infected T-cells. Although Tax is sufficient to upregulate the IRF-4 expression, leukemic cells are able to express IRF-4 in the absence of any Tax expression [42]. This is likely to be the consequence of, both, amplification and of point mutations in the *irf4* gene. This results in gain-of-function mutations within the DNA-binding domain of the protein [43]. Among them, K59R point mutation in *irf4*, which is commonly found in ATLL patients, leads to the expression of a more active IRF4 protein [44]. In addition, activation of the IRF4 could be driven by HBZ [37], whose expression is maintained in the ATLL cells [45]. Interestingly, HBZ did not directly induce the IRF4 expression but had an effect on the BATF3, the partner of IRF4 that is involved in regulation of a series of BATF3/IRF4-targeted genes that control cell proliferation and cell survival [37].

HAM/TSP patients are characterized by high IL-4 level in the serum [46] and an increased frequency of IL-4 expressing PBMCs [47], compared to asymptomatic carriers and healthy donors. In contrast, IL-4 expression within the CD4+ CD25+ CCR4+ (CC chemokine Receptor 4) T-cells, which is the phenotype of the HTLV-1-infected T-cells, is lower in the HAM/TSP patients, compared to healthy donors. This suggests that IL-4 is mainly expressed in other cell types, and could act in a paracrine manner to induce the proliferation of the HTLV-1-infected cells.

Finally, IL-4 could also be compensated by IL-13, since both cytokines signal through a complex receptor composed of IL-4R and IL-13R [48]. Interestingly, IL-13R is also upregulated in HTLV-1-infected cells [49]. Furthermore, IL-13 mRNA is highly expressed in the HTLV-1-infected CD4+ and CD8+ cell lines, and its expression is upregulated by the Tax protein, through NF-κB activation [50,51].

### 2.3. IL-6

IL-6-serum concentrations are higher in ATLL patients and HAM/TSP patients, compared to the levels observed both in asymptomatic carriers and healthy individuals [52,53]. IL-6 levels correlate with ATLL aggressiveness and a shorter survival rate [54]. IL-6 increase could result from its production by the HTLV-1-infected cells, themselves, since ATLL or HAM/TSP-derived IL-2-dependent T-cell lines, as well as CD8+ T cells or monocytes [55], are able to express IL-6 [56]. Tax expression from the HTLV-1-infected cell lines is required to induce IL-6R expression [57]. In addition, Tax also promotes the release of a soluble form of IL-6R (sIL-6R), whose level increases in the ATLL and HAM/TSP patients sera [57]. IL-6/sIL-6R complex can activate cells through their interaction with gp130, the IL-6 co-receptor [58]. Finally, IL-6 or IL-6/sIL-6R binding may also be dispensable, since constitutive activation of the STAT3, the downstream effector of IL-6 signaling, has been reported in the HTLV-1 transformed cell lines [57].

## 3. Paradoxical Functions of the IFN-I Antiviral Cytokine

The first cytokines to be induced, following a viral infection are those of innate immunity, including type I interferons (IFN-I), which have antiviral functions. The IFN-I family comprises several members—13 IFN-α subtypes, 1 IFN-β subtype, and less characterized cytokines, such as IFN-ε, -δ, -κ, -τ, -ω et -ζ [59]. IFN-α and -β production is induced through virus recognition by specific receptors that trigger a signaling cascade ending with the activation of IRF3 and IRF7 transcription factors [60]. Then, binding of IFN-I members to their receptor (IFNAR) and signaling through JAK/STAT [61] induces the expression of several ISGs (Interferon Stimulated Genes), with different antiviral functions [62].

The functions of IFN-I during HTLV-1 infection are not completely understood [63,64]. However, its use for therapy argues for a beneficial role, since a combination of IFN-α with zidovudine has better effect than classical chemotherapy, when provided to ATLL patients, except for those suffering from the lymphoma subtype [65]. Similarly, treatment of the HAM/TSP patients, with exogenous IFN-I, is associated with improvement of clinical symptoms [66,67,68,69] and with an inhibition of the T-cells’ spontaneous proliferation, as well as with a decrease in viral genes expression [70,71]. Nevertheless, how IFN-I promotes HTLV-1 control, *in vivo*, is still poorly understood. *In vitro*, recombinant IFN-α inhibits the infection by HTLV-1 of PBMCs, CBMCs (Cord Blood Mononuclear Cells), primary T-cells, and Jurkat T-cells [72,73], in a dose-dependent manner, notably through the expression of the ISG PKR (Protein Kinase R). This leads to a decrease in the HTLV-1 proteins expression and to the release of HTLV-1 particles by the newly infected T-cells [73,74]. Interestingly, IFN-I treatment of dendritic cells does not prevent their infection by HTLV-1 [3]. This suggests that the IFN-I-mediated control of HTLV-1 infection might be cell-type dependent. In contrast, the IFN-α treatment of the HTLV-1 transformed T-cell lines does not induce a reduction of viral proteins expression [74], suggesting that other ISGs can compete with PKR, or that the IFN-I response is inefficient when chronic infection is established. Indeed, the ISG ADAR1 (Adenosine Deaminase Acting on RNA 1) was shown to have a proviral role, by counteracting the PKR function [75], while the IFN-I signaling is abolished in the HTLV-1-infected cells [76,77,78] and the Tax-expressing cells [79].

Dysregulation of the IFN-I signaling does not seem to be associated with a decrease in cell surface expression of IFNAR in the HTLV-1-infected T-cells [78], but to an inhibition of JAK/STAT proteins phosphorylation [76,77,80]. This could partly be associated to the Tax-dependent upregulation of SOCS1 (Suppressor of Cytokine Signaling 1) [77,79], a known negative-regulator of the IFN-I signalization [62]. In addition, inhibition of IFN-I signaling might result from the inhibition of the transcriptional activity of the STAT proteins [68]. As reported for other viral infections [81], HTLV-1 infection is also expected to control the IFN-I production in infected T-cells. Nevertheless, how IFN-I response is induced or regulated during the HTLV-1 infection is still poorly understood. Tax abrogates IRF7 phosphorylation and transcriptional functions [82,83], but its effect on the IRF3 activation is more controversial. While some studies showed that Tax blocks the IRF3 phosphorylation and, thus, its transcriptional activity [77,82,84,85], others suggested a Tax-dependent induction of IRF3 phosphorylation, thus, enhancing its activity [86,87]. These discrepancies are likely linked to the different levels of Tax expression, in the different cellular models used in these studies.

If HTLV-1 infection can impact IFN-I production and signaling, this virus can also be recognized by several sensors, such as STING (Stimulator of Interferon Genes), IFI16 (Interferon-γ Inducible protein 16), and Ku70 [88,89,90], in the infected cells and by TLR-7 in plasmacytoid dendritic cells [91], which leads to the induction of IFN-I production, thus, potentially contributing to the HTLV-1 infection control. However, it is worth noting that the ATLL and HAM/TSP patients present a depletion of plasmacytoid dendritic cells [92,93,94], further supporting the role of IFN-I in the control of the HTLV-1 infection. While the PBMCs from the ATLL patients have a decreased ability to produce IFN-I, after triggering [92], a higher IFN-I signature is observed in the HAM/TSP patients, but not in the asymptomatic carriers [71]. This suggests that IFN-I response might be related to pathogenesis. Although IFN-I seems efficient to control the viral replication *in vitro* and to treat some HTLV-1 induced symptoms *in vivo*, the precise mode of action of this cytokine, as well as how and whether it could be induced in patients, needs further investigations.

## 4. Cytokines Promoting the HTLV-1-Induced Diseases Development

### 4.1. Cytokine Signature in HAM-TSP

Compared to healthy donors or asymptomatic carriers, the HAM/TSP patients are characterized by elevated levels of pro-inflammatory cytokines, such as IL-4, IL-6, IL-8, IFN-γ, and TNF-α (Tumor Necrosis Factor-α) in their plasma [95,96]. T-cells from the HAM/TSP patients spontaneously express IFN-γ, TNF-α, IL-6, and IL-1β [56,97], whose increased levels in the CSF [98] has been suggested to promote inflammatory immune responses in the HAM/TSP patients [99] (Figure 1B). Among these cytokines, IFN-γ has been described as the main driver for the induction of inflammation. It is worth noting that the HAM/TSP patients are characterized by an elevated IFN signature [71]. Thus, a chronic IFN-γ-stimulation of the immune cells, including the CTLs (cytotoxic T-cells) *in vivo*, could be deleterious for neural tissues [11,17].

In addition, IFN-γ favors the CD4+ T-cells migration, from blood to the central nervous system (CNS) [100], a characteristic of the HAM/TSP patients [101]. In addition, T-cells infiltration in the CNS might be increased because of permeability changes of the blood-brain barrier, due to tight junctions disruption between the human brain endothelial cells. This is induced either by the IL-1α and the TNF-α cytokines secreted by the HTLV-1-infected T-cells [102], or after the HTLV-1 endothelial cells infection [103]. Infiltration of the HTLV-1-infected CD4+ T-cells and CD8+ T-cells in neural tissues and in the CSF [14,15] leads to an increased proviral load (PVL) in the CSF, compared to the blood compartment [16]. This contributes to demyelination events within the CNS [17] (Figure 1B).

Other pro-inflammatory mediators, such as MIP-1α (Macrophage inflammatory protein-1α) and RANTES (Regulated on Activation, Normal T-cell Expressed and Secreted), are spontaneously produced by the PBMCs obtained from the HAM/TSP and could further contribute to the global inflammation process [104]. Of note, elevated serum levels of the immunomodulator IL-10 have also been observed in the HAM/TSP patients [55]. In contrast, in the asymptomatic carriers, IL-10 expressing CTL might counterbalance the induced-inflammation linked to the TNF-α production by monocytes. This effect is lost in the HAM/TSP patients, probably because of an increased IFN-γ-expressing CTL frequency [105]. Correction of the immunological imbalance with IL-10 up regulation has been observed in HAM/TSP patients, upon therapeutic intervention. This was correlated with clinical improvement [106].

Finally, elevated levels of neopterin in the plasma and the CSF of the HAM/TSP patients [107] could contribute to the neurotoxic events occurring in these patients, since the CSF-associated neopterin levels positively correlate to the severity of the clinical symptoms [108,109].

### 4.2. The Interplay between IFN-γ and CXCL10 in the Model of the HAM/TSP Development

Once infiltrated in the CNS, IFN-γ-producing HTLV-1-infected CD4+ T-cells induce CXCL10 (C-X-C motif chemokine Ligand 10) production by the astrocytes [101,110]. Probably linked to this, CXCL9 and CXCL10 concentrations are elevated in the HAM/TSP patients [95,111]. CXCL9 and CXCL10 associated levels in the CSF, correlate with the disease progression [109] and the PVL values [111]. Consequently, these two chemokines have been proposed to be markers of the HAM/TSP severity [109]. CXCL9 and CXCL10 are chemokines that are able to recruit the CXCR3 (C-X-C chemokine Receptor 3) expressing cells. CXCR3 is expressed by the IFN-γ producing CD4+ T-cells [112]. Thus, CXCL9 and CXCL10 expression promotes the positive feedback loop of the CXCR3+ T-cells recruitment in neural tissues, resulting in the maintenance of CNS chronic inflammation [101] (Figure 1B).

Astrocytes from the HAM/TSP patients constitute the cellular targets of HTLV-1 infections *in vivo* [113,114]. As such, through the expression of viral proteins, and specially Tax, they may be targeted by the HTLV-1-specific CTLs, and this could exacerbate the neural tissue damages [115,116]. Finally, Tax expression in astroglioma or astrocytoma cells leads to the expression of several pro-inflammatory cytokines, such as TNF-α, IL-1α, IL-1β, and IL-6 [117]. It has been suggested that secretion of these cytokines by microglia cells or monocytes could contribute to the HAM/TSP pathogenesis.

## 5. Cytokine Signature in ATLL

### 5.1. Low IFN-γ Expression

IFN-γ expression has been reported in some HTLV-1-infected T-cell lines [40,56,118,119]. It seems to be associated with Tax expression [120,121]. However, downregulation of Tax expression *in vivo*, and especially in leukemic cells [122], correlates with the lack of IFN-γ expression in CD4+ T-cells, freshly isolated from these patients [97]. This lack of IFN-γ production seems, therefore, likely linked to ATLL development, a hypothesis further supported by the observation that Tax-transgenic mice deficient of the IFN-γ expression, develop tumors [123]. This lower IFN-γ production could be associated with a decreased functionality of the CTLs, in combination with the elevated levels of IL-10, as described below (Figure 1C).

### 5.2. TGF-β Expression and Treg Development

*In vitro* culture of fresh ATLL cells and long-term ATLL T-cell lines, constitutively express TGF-β (Transforming Growth Factor-β) mRNA and secrete TGF-β [97,124,125], although TGF-β levels in the serum of ATLL patients are not significantly different from that of healthy donors [53]. Surprisingly, the TGF-β, known as an inhibitor of cell proliferation [126], does not prevent the spontaneous proliferation of the HTLV-1-infected T-cells [127]. While Tax is able to induce TGF-β production, by activating its gene promoter [124], several studies have also reported that Tax inhibits the TGF-β signaling, through reduction of the TGF-β receptor II expression, of Smad3/4 complex formation and its DNA binding on the TGF-β responsive elements, as well as of the recruitment of p300 by Smad proteins [128,129,130,131]. In contrast, HBZ overcomes the negative impact of Tax on the TGF-β signaling by favoring Smad3 and p300 interactions [132]. This could recapitulate an intact TGF-β signaling in leukemic cells, in which Tax expression is frequently silenced [133].

Apart from regulating cells proliferation, TGF-β signaling induces Foxp3 expression in peripheral CD4+ T-cells [134] and promotes regulatory T-cells responses. Interestingly, ATLL patients have a number of blood-circulating Foxp3+ CD4+ T-cells, higher than healthy donors [135,136]. Furthermore, a positive correlation between Foxp3+ CD4+ T-cells frequency, among the PBMCs and the CCL22 plasmatic levels, has been reported in the asymptomatic carriers and in chronic ATLL patients [136]. This cytokine induces the Foxp3+ CD4+ T-cells migration and sustains their viability [136]. These cells, obtained from patients with acute or chronic ATLL, are able to inhibit the CD4+ CD25+ T-cells autologous proliferation. They also decrease the rate of CTL-dependent lysis of the autologous Tax+ CD4+ T-cells [137]. Defining the Treg function in ATLL pathogenesis, therefore, remains delicate. On one hand, Treg may limit ATLL development by inhibiting leukemic cells proliferation. On the other hand, Treg could inhibit the rate of the infected T-cells lysis by the CD8+ T-cells, thus, contributing to the escape of the HTLV-1-infected cells from adaptive immune responses.

### 5.3. IL-10 and T-Cell Exhaustion

Sera from ATLL patients contain increased levels of IL-10, compared to the asymptomatic carriers, the HAM/TSP patients, or the healthy donors [53,55,138]. Interestingly, IL-10 levels are even higher in patients with the aggressive subtypes of ATLL, than in patients with indolent ATLL, therefore, associating IL-10 levels to a poor prognosis [53,138]. HTLV-1-infected T-cell lines and freshly isolated ATLL cells are also able to release IL-10 in the supernatant [39,56,139], and the absolute frequency of IL-10 producing cells is higher in the ATLL patients, than in the asymptomatic carriers [138]. IL-10 expression is induced by Tax or HBZ [138,140]. IL-10 promotes cell survival, cell proliferation, and even IL-10 production in an autocrine/paracrine manner [56]. In ATLL patients, high levels of IL-10 have been associated with the malignant proliferation of HTLV-1-infected cells [55]. Along with the HTLV-1-infected CD4+ T-cells, IL-10 could also be expressed by the HTLV-1-infected macrophages [141], dendritic cells [140], or Treg [142], whose levels are increased in ATLL patients.

IL-10 has been reported to contribute to the immune escape of several other viruses and to deplete the CD8+ T-cell responses [143], notably, through the induction of the exhaustion marker PD-1 on T-cells [144]. Frequencies of the PD-1 expressing CD4+ and CD8+ T-cells are increased in the ATLL patients [145,146]. Furthermore, in asymptomatic carriers, PD-1+ Tax-specific CTLs frequency positively correlates with the HTLV-1 proviral load (PVL) in the PBMCs, and inversely, with the production of IFN-γ and TNF-α, after their stimulation with cognate peptides [147]. These results could be associated to the decreased frequency, diversity, and function of the anti-HTLV-1 Tax-specific CD8+ T-cells observed in the ATLL patients, and could favor the ATLL pathogenesis [148]. Indeed, interfering with the PD-1 triggering, enhances the IFN-γ and TNF-α production by the HTLV-1 specific CD8+ T-cells [145]. High serum IL-10 level has also been observed in HAM/TSP patients [55], although PD-1 expression on the CTLs from the HAM/TSP patients has not been reported, yet. In addition to an increased ratio of the CD8^+^IFN-γ^+^/CD8^+^IL-10^+^ T-cells in the HAM/TSP, the immunosuppressive effect of IL-10 could also be counterbalanced by the elevated levels of the IFN-γ in the CSF, from the HAM/TSP patients.

## 6. Conclusions

Cytokine network supporting viral persistence and eventually viral-induced diseases is summarized in Figure 1. Several interleukins are involved in the HTLV-1-infected T-cells proliferation and survival, thus, contributing to viral persistence in infected individuals. Alterations of cytokines signaling, within the HTLV-1-transformed T-cells, lead to a cytokine-independent growth, which could partly contribute to the ATLL development. Furthermore, viral persistence could be enhanced by the HTLV-1 properties to dysregulate the early IFN-I responses, *in vivo*. Dysregulation of the immune cytokine networks in the HAM/TSP and ATLL patients, is characterized by an exacerbated expression of the pro-inflammatory or immunosuppressive cytokines, respectively, which have detrimental effects on overall immune responses. In addition to the elevated levels of these cytokines in the fluids of patients, it is more likely that direct dysregulations of the balance between functionally opposite cytokines, such as IFN-γ/IL-10 balance, contributes to the pathogenesis [96,149]. A complex network of cytokines could first generate a microenvironment, advantageous to the persistence of the HTLV-1-infected cells and, then, could contribute to the HTLV-1-induced diseases development.
